# Live Birth Sex Ratio after *In Vitro* Fertilization and Embryo Transfer in China - An Analysis of 121,247 Babies from 18 Centers

**DOI:** 10.1371/journal.pone.0113522

**Published:** 2014-11-20

**Authors:** Zhiqin Bu, Zi-Jiang Chen, Guoning Huang, Hanwang Zhang, Qiongfang Wu, Yanping Ma, Juanzi Shi, Yanwen Xu, Songying Zhang, Cuilian Zhang, Xiaoming Zhao, Bo Zhang, Yuanhua Huang, Zhengyi Sun, Yuefan Kang, Riran Wu, Xueqing Wu, Haixiang Sun, Yingpu Sun

**Affiliations:** 1 Reproductive Medicine Center, First Affiliated Hospital of Zhengzhou University, Zhengzhou, People's Republic of China; 2 Reproductive Medicine Center, Nanjing Drum Tower Hospital, Nanjing University, Nanjing, People's Republic of China; 3 Center for Reproductive Medicine, Provincial Hospital Affiliated to Shandong University, Jinan, People's Republic of China; 4 Chongqing Reproductive and Genetics Institute, Chongqing Obstetrics and Gynecology Hospital, Chongqing, People's Republic of China; 5 Reproductive Medicine Center, Tongji Hospital, Huazhong University of Science and Technology, Wuhan, People's Republic of China; 6 Reproductive Medicine Centre, Women and Children Hospital of Jiangxi Province, Nanchang, People's Republic of China; 7 Reproductive Medicine Center, First People's Hospital of Yunnan Province, Kunming, People's Republic of China; 8 Reproductive Medicine Center, Maternal and Child Health Care Hospital of Shaanxi Province, Xi'an, People's Republic of China; 9 Reproductive Medicine Center, First Affiliated Hospital of Sun Yat-Sen University, Guangdong, People's Republic of China; 10 Reproductive Medicine Center, Sir Run Run Shaw Hospital, School of Medicine, Zhejiang University, Hangzhou, People's Republic of China; 11 Reproductive Medicine Center, Henan Provincial People's Hospital, Zhengzhou, People's Republic of China; 12 Reproductive Medicine Center, Renji Hospital, Shanghai Jiaotong University, Shanghai, People's Republic of China; 13 Reproductive Medicine Center, Maternal and Child Health Hospital of Guangxi Zhuang Autonomous Region, Nanning, People's Republic of China; 14 Reproductive Medicine Center, Affiliated Hospital of Hainan Medical College, Haikou, People's Republic of China; 15 Department of Obstetrics and Gynecology, Peking Union Medical College Hospital, Peking Union Medical College and Chinese Academic of Medical Sciences, Beijing, People's Republic of China; 16 Reproductive Medicine Center, Maternal and Children's Health Hospital of Fujian Province, Fuzhou, People's Republic of China; 17 Reproductive Medicine Center, Boai Hospital of Zhongshan, Zhongshan, People's Public of China; 18 Reproductive Medicine Center, Women and Children's Hospital of Shanxi Province, Taiyuan, People's Republic of China; State Key Laboratory of Reproductive Biology, Institute of Zoology, Chinese Academy of Sciences, China

## Abstract

In order to study the impact of procedures of IVF/ICSI technology on sex ratio in China, we conducted this multi-center retrospective study including 121,247 babies born to 93,895 women in China. There were 62,700 male babies and 58,477 female babies, making the sex ratio being 51.8% (Male: Female  = 107∶100). In univariate logistic regression analysis, sex ratio was imbalance toward females of 50.3% when ICSI was preformed compared to 47.7% when IVF was used (*P*<0.01). The sex ratio in IVF/ICSI babies was significantly higher toward males in transfers of blastocyst (54.9%) and thawed embryo (52.4%) when compared with transfers of cleavage stage embryo (51.4%) and fresh embryo (51.5%), respectively. Multiple delivery was not associated with sex ratio. However, in multivariable logistic regression analysis after controlling for related factors, only ICSI (adjusted OR = 0.90, 95%CI: 0.88–0.93; *P*<0.01) and blastocyst transfer (adjusted OR = 1.14, 95% CI: 1.09–1.20; *P*<0.01) were associated with sex ratio in IVF/ICSI babies. In conclusion, the live birth sex ratio in IVF/ICSI babies was influenced by the use of ICSI, which may decrease the percentage of male offspring, or the use of blastocyst transfer, which may increase the percentage of male offspring.

## Introduction

In humans, it is estimated that the primary sex ratio after conception may be as high as 170∶100 [1]. However, since spontaneous abortion and preterm delivery are more likely to happen to mothers carrying male babies [2], the sex ratio at birth in humans, which is known as the secondary sex ratio (SSR), is around 105∶100 [3]. Studies have shown that SSR is associated with many biologcial and enviromental factors. It is shown that older materanl age, high stress, social factors reduce the SSR in natural conception [4], [5], [6], [7].

Sicne the first *in vitro* fertilization (IVF) baby was born in 1978 in England, assisted reproductive techonology (ART) has rapidly developed all over the world. Concerns about whether IVF/ICSI has any impacts on SSR have led scholars to study SSR in IVF/ICSI babies [8], [9], [10]. Even though no conclusions has been made, there is a trend of a higher SSR in ART babies following IVF compared with those conceived after intracytoplasmic sperm injection (ICSI). In addition, many studies also demonstrated that the SSR was skewed in favor of males born after blastocyst transfer compared to those born after cleavage-stage embryo transfer in the same time period [8], [9], [11], [12], [13].

Similar studies have shown that large sample size, data from different countries and regions are needed to study SSR in IVF/ICSI offspring. A careful review of the literature has shown that SSR in babies following IVF/ICSI was reported in United States, Australia and New Zealand, Japan, etc [8], [9], [10]. However, no studies to date have reported the sex ratio of offspring conceived after IVF/ICSI in China, which has the largest population in the world. The aim of this large sample size study was to report the SSR in babies born after IVF/ICSI in China, and to evaluate the impact of ART procedures on SSR.

## Materials and Methods

All the babies born after IVF/ICSI in this retrospective study were recruited from 18 reproductive medicine centers in China (Two centers from Northern China, 4 centers from Central China, 3 centers from Southern China, 4 centers from Eastern China, 2 centers from Northwestern China, and 3 centers from Southwestern China). Since no previous reports has been published concerning SSR in IVF/ICSI babies in China, this study included all the offspring after IVF/ICSI from each center from January 2002 to December 2012. Those conceived by sperm donation cycles, oocyte donation cycles, or pre-implantation genetic diagnosis cycles were excluded. Institutional Review Board (IRB) approval was obtained from First Affiliated Hospital of Zhengzhou University. Written consent forms were obtained from patients in some centers. However, all the patients were undergoing routine IVF/ICSI treatment, and patient information was totally anonymous in our analysis.

The data variables included in the study were live birth cycles, the babies' number and sex, insemination method (IVF or ICSI), stage of embryo transferred (cleavage stage embryo or blastocyst), type of embryo (fresh or thawed), and type of birth (singleton or multiple delivery). Cleavage stage embryo was defined as a day-2 or day-3 embryo. Blastocyst transfer was a transfer of a day-5 to day-7 embryo. Live birth was defined as any birth event in which at least one baby is born alive [14]. Multiple delivery was defined as twin or higher order pregnancy. SSR was defined as: Number of male babies/Number of total babies.

For univariate analysis of categorical variables, chi-square tests were used. Simple binary logistic regression was used to generate the crude odds ratio (OR), 95% of confidence interval and *P* value for the final univariate analysis. Multivariable binary logistic regression was used to generate the adjusted OR. A *P* value <0.05 was considered to be significant. SPSS 17.0 (SPSS Inc., Chicago, IL, USA) was used for analysis.

## Results

In total, 18 reproductive medicine centers were included into this study. The study population included 121,247 babies born to 93,895 women (File S1). There were 62,700 male babies and 58,477 female babies. The SSR in IVF/ICSI babies was 51.8% (Male: Female  = 107∶100). The SSRs of the 6 regions were different. The SSR of Central China was the highest (52.6%, M:F = 111∶100); and the SSR of Northwestern China was the lowest (49.3%, M:F = 97∶100) (Table 1).

**Table 1:** Sex ratio of live birth offspring by different ART procedures and type of birth.

	Total live birth	Female	Male	M:F	SSR (%)
**Fertilization procedure** *					
IVF	79,606	37,949	41,657	110∶100	52.3%
ICSI	31,276	15,721	15,555	99∶100	49.7%
**Stage of transferred embryo**					
Cleavage-stage embryo	107,967	52,489	55,478	106∶100	51.4%
Blastocyst	13,280	5,988	7,292	122∶100	54.9%
**Type of embryo**					
Fresh ET	84,649	41,041	43,608	106∶100	51.5%
Thawed ET	36,598	17,436	19,162	110∶100	52.4%
**Type of birth**					
Singleton	66,676	32,107	34,569	107∶100	51.8%
Multiple birth	54,571	26,370	28,201	107∶100	51.7%
**Location of fertility center**					
Northern China	20487	9894	10593	107∶100	51.7%
Central China	35989	17052	18937	111∶100	52.6%
Southern China	11231	5390	5841	108∶100	52.0%
Eastern China	24522	11872	12650	107∶100	51.6%
Northwestern China	7837	3976	3861	97∶100	49.3%
Southwestern China	21181	10293	10888	106∶100	51.4%

IVF =  *in vitro* fertilization; ICSI =  intracytoplasmic sperm injection; ET =  embryo transfer; ART =  assisted reproductive technology; SSR =  secondary sex ratio.

*Insemination method was not clear in 10,365 cycles.

Firstly, the sex ratio was analyzed according to different ART procedures and singleton or multiple delivery (Table 1). There was a sex ratio imbalance toward females of 50.3% (M: F = 99∶100) when ICSI was performed, compared to 47.7% (M: F = 110∶100) when IVF was used (*P*<0.01). In contrast, the SSR was significantly higher towards males in blastocyst transfers compared with that in cleavage stage embryo transfers (54.9% vs. 51.4%, *P*<0.01). In addition, we also found a sex ratio imbalance towards males of 52.4% in thawed ETs compared with 51.5% in fresh ETs (*P*<0.01). However, the SSR in singleton and multiple delivery were similar (51.8% vs. 51.7%, *P*>0.05).

Results of multivariable logistic regression analysis were showed in Table 2. After adjusting for confounding factors, ICSI (adjusted OR = 0.90, 95%CI: 0.88–0.93; *P*<0.01) and blastocyst transfer (adjusted OR = 1.14, 95% CI: 1.09–1.20; *P*<0.01) were still significantly associated with SSR in IVF/ICSI babies. However, the association between fresh/thawed transfer and SSR was not significant after adjusting for other factors (adjusted OR = 0.99, 95% CI: 0.96–1.02; *P*>0.05).

**Table 2:** Factors affecting SSR in ART babies by logistic regression analysis.

	Crude OR (95% CI)	Adjusted OR (95% CI)
**Fertilization procedure**	0.90** (0.88–0.93)	0.90**(0.88–0.93)
*0 = IVF; 1 = ICSI*		
**Stage of embryo**	1.15**(1.11–1.20)	1.14**(1.09–1.20)
*0 = cleavage stage embryo; 1 = blastocyst*		
**Type of embryo**	1.03** (1.01–1.06)	0.99 (0.96–1.02)
*0 = fresh embryo; 1 = thawed embryo*		
**Type of birth**	0.99 (0.97–1.02)	—
*0 = singleton; 1 = multiple birth*		

**P<0.001.

IVF =  *in vitro* fertilization; ICSI =  intracytoplasmic sperm injection; ET =  embryo transfer; OR =  odds ratio; CI =  confidence interval; ART =  assisted reproductive technology; SSR =  secondary sex ratio.

**Figure 1:**
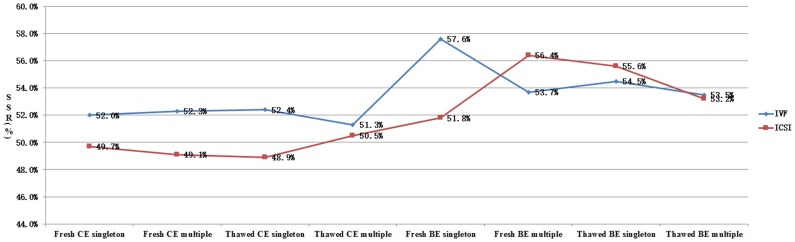
Effect of insemination method (IVF/ICSI) on SSR. SSR =  secondary sex ratio; IVF =  in vitro fertilization; ICSI =  intracytoplasmic sperm injection; CE =  cleavage stage embryo; BE =  blastocyst embryo.

**Figure 2:**
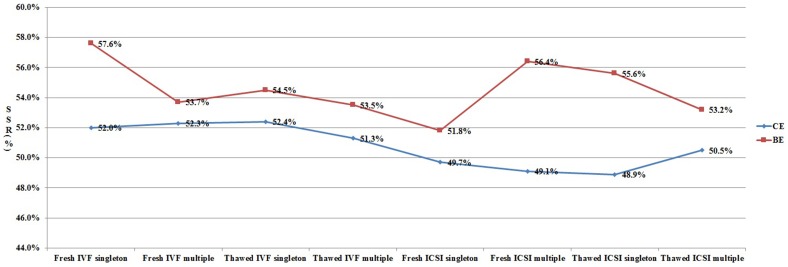
Effect of stage of embryo transferred (CE/BE) on SSR. SSR =  secondary sex ratio; IVF =  in vitro fertilization; ICSI =  intracytoplasmic sperm injection; CE =  cleavage stage embryo; BE =  blastocyst embryo.

**Figure 3:**
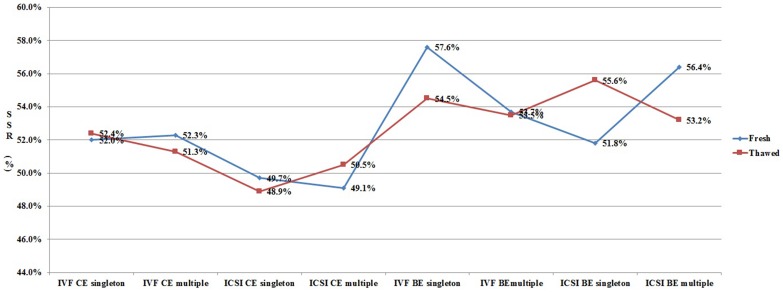
Effect of type of embryo transferred (fresh/thawed) on SSR. SSR =  secondary sex ratio; IVF =  in vitro fertilization; ICSI =  intracytoplasmic sperm injection; CE =  cleavage stage embryo; BE =  blastocyst embryo.

**Figure 4:**
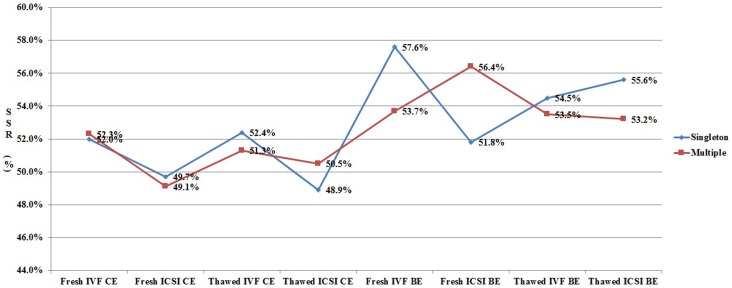
Effect of type of birth (singleton/multiple delivery) on SSR. SSR =  secondary sex ratio; IVF =  in vitro fertilization; ICSI =  intracytoplasmic sperm injection; CE =  cleavage stage embryo; BE =  blastocyst embryo.

In order to clarify the different effects of ART procedures on live birth sex ratio, we analyzed all the data from each subgroup (Fig. 1–3). As shown in Figure 1, for patients undergoing cleavage stage embryo transfer, the SSR was significantly higher in IVF group when compared with that in ICSI group. However, the SSR in IVF group was not always higher than that in ICSI group for patients undergoing blastocyst transfer. Figure 2 shows the effect of blastocyst transfer on SSR. Irrespective of insemination methods, type of embryo transferred, or type of birth, the SSR in blastocyst transfer group was higher than that in cleavage stage transfer group. Figure 3 and Figure 4 describe the relationship between SSR and type of embryo transferred, type of birth, respectively. There was no obvious regularity in either of these two figures, indicating that SSR was not related with type of embryo transferred, or type of birth.

## Discussion

Recently, IVF/ICSI has been developed rapidly, and clinical pregnancy and live birth rates are stable. It is shown that around 130,000 IVF/ICSI babies were born in the year of 2005 in America, which accounts for 1% new born babies in the whole country [9]. Thus, it is urgent and important to monitor the safety of IVF/ICSI.

### SSR in babies born after IVF/ICSI

As early as 2000, scholars from French and Australia reported the SSR in IVF/ICSI babies [12], [13]. However, since most of those studies were small sample size and were from Europe, the conclusions were not convincing. In 2014, a study from Japan including more than 40,000 IVF/ICSI babies has shown that SSR was 52.7% (111∶100). In addition, another large sample size from Australia and New Zealand showed that SSR in IVF/ICSI babies was 51.3% (105∶100). This current study which included more than 120,000 babies showed that SSR in China was 51.8% (107∶100), which was similar with previous studies. SSR in natural conception and delivery was around 105–110∶100, thus we conclude that SSR is not altered in IVF/ICSI babies compared with natural conception [3].

The current study also analyzed SSRs from different regions of China. According to the location of the 18 centers, all IVF/ICSI babies were divided into 6 regions: Northern China, Central China, Southern China, Eastern China, Northwestern China, and Southwestern China. The SSRs were different among these 6 regions. Thus, here we advocate that it is necessary to include multiple centers from different regions in such studies in the future.

### The effect of insemination method on SSR in IVF/ICSI babies

ICSI, which has been mainly used to treat male factor infertility, is now widely used all over the world. The use of ICSI in the United States has increased from 11.0% of ART cycles to 57.5% of cycles between 1995 and 2004 [9]. Thus, more and more studies have been conducted to explore the safety of ICSI [15], [16]. However, there were few large sample size studies concerning the effect of ICSI on SSR. Data from other counties have shown that the use of ICSI significantly reduces the proportion of male babies compared with the use of IVF (48.8% versus 51.4% in America; 50.0% versus 53.0% in Australia and New Zealand), which is consistent with our results [8], [9].

However, since the SSR in IVF/ICSI could be affected by other factors, we explored the association between SSR and insemination methods in subgroups (Figure 1). Our results showed that ICSI reduces SSR only in cleavage stage embryo transfer cycles, but not in blastocyst transfer cycles. We speculate that the reduction of SSR by ICSI may be compensated by the increase of SSR by blastocyst transfer, making the SSR in ICSI cycles with blastocyst transfer not being altered.

Currently, there is no conclusion why the use of ICSI reduces SSR. ICSI is mainly used in patients with male factor infertility, of whom the spermatogenic function is usually compromised. It may be possible that the percentage of Y-bearing sperm is also reduced in those patients [17]. However, other studies have shown that the use of ICSI in patients with normal spermatogenic function (unexplained infertility) also reduces the SSR. An increase in Y chromosome abnormalities that directly affect embryogenesis would be suspected [8].

### The effect of stage of embryo transferred on SSR in IVF/ICSI babies

Compared with cleavage stage embryo transfer, blastocyst transfer significantly increases implantation rate, and reduces multiple pregnancy rate [18], [19]. However, recent studies have shown that blastocyst transfer increase the risk of preterm birth, and may increase birth defects [20], [21]. In addition, the present study has shown that blastocyst transfer significantly increases SSR compared with cleavage stage embryo transfer. In the 1990s, several studies with small sample had shown that blastocyst transfer may alter SSR in IVF/ICSI babies [13], [22], [23]. A recent meta-analysis and other large sample size studies also showed blastocyst transfer is associated with sex ratio imbalance toward males [24], [25]. We here summarized three studies with more than 10,000 IVF/ICSI babies in the last 5 years, and found that in the 42,587 babies after blastocyst transfer, there were 22,813 males and 19,774 female, making the SSR was 53.4%. However, in the 33,845 babies after cleavage stage embryo transfer, which included 16,814 males and 17,031 females, the SSR was 49.7%. The SSR for cleavage stage embryo transfer and blastocyst transfer were 54.9% and 51.4%, respectively [8], [9], [10].

It is worthwhile to note that we also explored the relationship between stage of embryo transferred and SSR in subgroups (Figure 2). Compared with cleavage stage embryo transfer, blastocyst transfer increases SSR, and this effect is independent of other factors.

### The effect of type of embryo transferred on SSR in IVF/ICSI babies

In the present study, we also explored the effect of frozen-thawed embryo transfer (FET) on SSR. To date, very few studies are reporting the difference of SSR in fresh embryo transfer and FET, and the results are controversial [22], [26]. Our preliminary univariate logistic regression analysis showed that FET increases SSR (COR = 1.03,95% CI: 1.01–1.06,P<0.01). However, in multivariate logistic regression analysis after controlling for related factors, SSR was not related with FET. Subgroup analysis also showed that SSR was not related with FET (Figure3). In China, blastocyst transfers are usually in FET cycles in most of the reproductive medicine centers. It is maybe the blastocyst transfer, but not FET, that is responsible for the alteration of SSR toward males.

### The effect of type of birth on SSR in IVF/ICSI babies

In most of the previous studies, only singleton or single embryo transfer cycles were included into analysis, because monozygotic twins in multiple pregnancies may also have effect on SSR [24]. However, the present study showed that multiple delivery has no effect on SSR by both logistic regression analysis and subgroup analysis. The SSRs in singleton and multiple delivery are comparable (51.8% versus 51.7%). Thus, it is also necessary to include multiple delivery into SSR analysis in future studies.

To the best of our knowledge, this is the first study reporting SSR in babies born after IVF/ICSI in China. Even though with large sample size from 18 centers, the present study indeed has several limitations. Since there is no standard ART data reporting system in China, currently it is impossible to collect as much information, such as mothers' basic characteristics, as we want. Even though basic characteristics of 5,790 patients with singleton birth were obtained from only one center (Table S1), and there indeed were no differences in age, infertility duration, number of embryos transferred, ect between mothers giving birth to a boy and those giving birth to a girl; however, it would be better if we could get these detailed information from all the 18 centers include into this study. Second, we included all IVF/ICSI babies in the last 10 years, which is a relative long period, into this study. Some IVF/ICSI procedures or embryo culture medium are changing in short times, this may also bring some bias into our analysis. Another reason why we should be careful with our results is that, blastocyst transfer has only been widely used in China in recent years. There were 13,280 babies (11.0% in total) born after blastocyst transfer in this study. Studies with larger sample size are needed in the future.

Taken together, the present study including more than 120 thousands IVF/ICSI babies from 18 reproductive medicine centers in China showed that the overall SSR in IVF/ICSI is 51.8% (107∶100), which is similar with that in natural conception. Compared with the use of IVF, the use of ICSI reduces SSR in cleavage stage embryo transfer cycles, but not in blastocyst transfer cycles. However, blastocyst transfer increases SSR compared with cleavage stage embryo transfers, and this effect is independent of other factors. Neither FET nor multiple delivery has effect on SSR.

## Supporting Information

Table S1 **Basic characteristics of 5,790 mothers with singleton birth in one center.**
(DOC)

File S1 **121,247 IVF/ICSI babies from 18 centers in China.** IVF-ET  =  *in vitro* fertilization and embryo transfer; ICSI-ET  =  intracytoplasmic sperm injection and embryo transfer; Cleavage stage  =  Day 2 or day 3 embryo transfer; Blastocyst  =  Day 5 or day 6 embryo transfer.
(DOC)
