# Supplementary material for: Live Birth Sex Ratio after In Vitro Fertilization and Embryo Transfer in China - An Analysis of 121,247 Babies from 18 Centers
Source: PLoS One. 2014 Nov 20;9(11):e113522. doi: 10.1371/journal.pone.0113522 (PMC4239103; doi:10.1371/journal.pone.0113522)
Supplement: Table S1 — Basic characteristics of 5,790 mothers with singleton birth in one center. (DOC) [file pone.0113522.s001.doc]

**Table S1. Basic characteristics of 5,790 mothers with singleton birth in** one center

|  | Mothers in Group A  N = 3039 | Mothers in Group B  N = 2751 | T | P |
| --- | --- | --- | --- | --- |
| Mother’s age (y) | 30.5±4.6 | 30.3±4.6 | 1.9 | 0.06 |
| Basal FSH (mIU/mL) | 7.5±3.4 | 7.6±3.9 | -0.8 | 0.39 |
| BMI (Kg/m^2^) | 22.3±3.1 | 22.3±3.0 | 1.2 | 0.24 |
| Infertility duration (y) | 4.4±3.2 | 4.5±3.2 | -1.0 | 0.32 |
| No. of embryos transferred | 2.1±0.6 | 2.1±0.6 | -1.3 | 0.21 |

Note: FSH, follicle stimulation hormone; BMI, body mass index; Data were presented as Mean±SD.

Group A: Mother who gives birth to a baby boy; Group B: Mother who gives birth to a baby girl.
