# Supplementary material for: Live Birth Sex Ratio after In Vitro Fertilization and Embryo Transfer in China - An Analysis of 121,247 Babies from 18 Centers
Source: PLoS One. 2014 Nov 20;9(11):e113522. doi: 10.1371/journal.pone.0113522 (PMC4239103; doi:10.1371/journal.pone.0113522)
Supplement: File S1 — 121,247 IVF/ICSI babies from 18 centers in China. IVF-ET = in vitro fertilization and embryo transfer; ICSI-ET = intracytoplasmic sperm injection and embryo transfer; Cleavage stage = Day 2 or day 3 embryo transfer; Blastocyst = Day 5 or day 6 embryo transfer. (DOC) [file pone.0113522.s002.doc]

**File S1. 121,247 IVF/ICSI babies from 18 centers in China**

|  | | **IVF-ET** | | | | **ICSI-ET** | | | | **Total** | | | |
| --- | --- | --- | --- | --- | --- | --- | --- | --- | --- | --- | --- | --- | --- |
|  |  | **Cleavage stage** | | **Blastocyst** | | **Cleavage stage** | | **Blastocyst** | | **Cleavage stage** | | **Blastocyst** | |
|  |  | **Female** | **Male** | **Female** | **Male** | **Female** | **Male** | **Female** | **Male** | **Female** | **Male** | **Female** | **Male** |
| **Fresh** | **Singleton** | **14814** | **16080** | **506** | **686** | **6257** | **6172** | **191** | **205** | **21071** | **22252** | **697** | **891** |
|  | **Multiple** | **13574** | **14902** | **216** | **251** | **5387** | **5188** | **96** | **124** | **18961** | **20090** | **312** | **375** |
| **Thaw** | **Singleton** | **4022** | **4433** | **1147** | **1373** | **1874** | **1795** | **378** | **473** | **7330** | **7738** | **3009** | **3688** |
|  | **Multiple** | **2992** | **3152** | **678** | **780** | **1301** | **1329** | **237** | **269** | **5127** | **5398** | **1970** | **2338** |
